# Optical Characteristics of Monochrome and Multilayer Fully Stabilized Zirconia Upon Sintered Cooling Speed

**DOI:** 10.1055/s-0043-1764233

**Published:** 2023-04-14

**Authors:** Pithiwat Uasuwan, Niwut Juntavee, Apa Juntavee

**Affiliations:** 1 Division of Biomaterials and Prosthodontics Research, Faculty of Dentistry, Khon Kaen University, Khon Kaen, Thailand; 2 Department of Prosthodontics, Faculty of Dentistry, Khon Kaen University, Khon Kaen, Thailand; 3 Division of Pediatric Dentistry, Department of Preventive Dentistry, Faculty of Dentistry, Khon Kaen University, Khon Kaen, Thailand

**Keywords:** color appearance, contrast, cooling, opalescence, translucency

## Abstract

**Objectives** Firing protocols influence optical properties of dental ceramics. Effects of varying cooling rates of monochrome and multilayer 5 mol% yttria-stabilized tetragonal polycrystalline (5YTZP) on optical properties are subjected for investigation.

**Materials and Methods** Ninety specimens (width, length, thickness = 10 × 20 × 2 mm) were prepared from monochrome (Mo: Cercon xt) and multilayer (Mu: Cercon xt ML with cervical (C) and incisal (I) zoning) 5YTZP. Specimens were sintered and randomly treated with three cooling rates ( *n* = 15/group): slow (S: 5°C/min), normal ( *N* : 35°C/min), and fast (F: 70°C/min). Color appearance (∆E _W_ ), color appearance difference (∆ *E*_diff_ ), translucency parameter (TP), contrast ratio (CR), and opalescence parameter (OP) were evaluated in CIEL*a*b* (Commission International de I'Eclairage) system. ∆ *E*_diff_ was achieved from the coordinate difference of specimen to VITA classic shade A2. Microstructures and compositions were evaluated by scanning electron microscope and energy dispersive spectroscopy. Monoclinic ( *m* ), tetragonal ( *t* ), and cubic ( *c* ) phases were investigated with X-ray diffraction.

**Statistical Analysis** An analysis of variance and Bonferroni multiple comparisons were determined for significant differences ( *p* < 0.05).

**Results** ΔE _W_ of MoF was highest (66.04 ± 1.86), while MuN-I was lowest (62.60 ± 0.86). TP and OP of MoS were highest at 2.85 ± 0.11, and 2.25 ± 0.10, while MuF-I was lowest at 2.16 ± 0.10 and 1.60 ± 0.12. CR of MuF-I was highest (0.948 ± 0.005), while MoS was lowest (0.936 ± 0.005). ΔE _diff_ of MoF was highest (3.83), while MuN-I was lowest (0.93). Limited grain growth and m-phase composition were indicated upon fast cooling. There were significant differences for all color parameters due to varied materials, cooling rates, and their interactions ( *p* < 0.05) except for interaction in ∆E _W_ and OP.

**Conclusions** Translucency of monochrome and multilayer 5YTZP were different, possibly due to colorant additives. Incisal layer of multilayer 5YTZP was perfectly matched with VITA shade. Increasing cooling speed resulted in smaller grain size, t-m transformation, and finally lower translucency and opalescence. Therefore, to achieve most favorable optical properties, slow cooling rate is recommended.

## Introduction

The development of computer technology has led to the emergence of a variety of dental ceramics to meet patients' needs. Dental ceramics, with their extraordinary physical properties, have been used for aesthetic purposes for many years. 1 Dental ceramics can be classified as glass matrix ceramics (e.g., lithium disilicate glass), polycrystalline ceramics (e.g., stabilized zirconia), and resin matrix ceramics. 2 Zirconia is a highly appealing ceramic material in restorative dentistry due to its excellent mechanical strength, fracture toughness, chemical properties, and dimensional stability. Moreover, the modulus of elasticity of zirconia is close to that of stainless steel alloy. 3 Zirconia is one of the polycrystalline ceramics that consist of three phases of crystal structure: monoclinic ( *m* ), tetragonal ( *t* ), and cubic ( *c* ). The monoclinic phase is presented at normal temperature and transforms to the tetragonal phase when the temperature reaches 1,170°C. Up to 2,370°C, the shift from the tetragonal phase to the cubic phase occurs, and this cubic phase remains unchanged until it reaches the 2,680°C melting point. 4 Zirconia's tetragonal and cubic phases can be stabilized at room temperature through the incorporation of oxide stabilizers. Yttrium oxide (Y _2_ O _3_ ) has been introduced as a stabilizer and the most widely used oxide. 4 The 3 mol% yttria-stabilized tetragonal polycrystalline (3YTZP) has primarily been manufactured, and the addition of 4 mol% and 5 mol% of yttrium oxide has also been recently reported. External stimulants such as humidity, stress, and heat could trigger the shift from the tetragonal to the monoclinic phase, resulting in a 4 to 5% expansion of the microstructure. 5 This volumetric change induces compressive stress to withstand the propagation of cracks, known as “transformation toughening,” 6 which gives stabilized zirconia its extraordinary strength. The opacity of yttria-stabilized tetragonal zirconia polycrystalline (YTZP) has to be taken into consideration, since it is composed of completely different crystal structures with different refractive indices, resulting in excessive light scattering and diffuse reflectance compared to glass matrix ceramics. 7 Because of this, zirconia was predominantly fabricated as a substructure for porcelain veneering to simulate the effect of light on natural teeth. However, the presence of delamination and chipping of overlay porcelain has been widely reported. 8 9 Monolithic zirconia was then implemented to overcome this issue, and techniques to improve its optical properties have been pursued. 9

Optical properties, including the perception of color, translucency, contrast, and opalescence, are the primary considerations for material selection, especially in the field of aesthetics. 10 Concerning the perception of color, the color appearance difference (∆ *E*_diff_ ) was applied to determine the level of color perception. A value of ∆ *E*_diff_ < 3 indicated “ *clinically indifferent,* ” ∆ *E*_diff_ = 3-5 indicated “ *clinically acceptable,* ” and ∆ *E*_diff_ >5 indicated “ *clinically unacceptable.* ” 11 Translucency was described by the degree of light transmission through an object. The greater the light transmission, the higher the translucency. 12 In other words, the condition that exists between total opacity and transparency is known as translucency. This optical property is reflected by the translucency parameter (TP) and contrast ratio (CR) 13 ; the material with greater translucence would present a higher TP value and lower CR value since they are negatively correlated. 14 The grain size, crystal structure, color additives, and porosity were reported to affect the light trajectory. 13 The restorative material should appear blueish when light is reflected off it and have an orange appearance when light transmits through it. This phenomenon is known as “opalescence” and is important for closely simulating the natural appearance of enamel structure. It is determined by the opalescence parameter (OP), 14 and human enamel has an OP value of 19.8 to 27.6. 15 There have been many attempts to improve the translucency of monolithic zirconia including the modification of the sintering parameter, the modification of alumina content, and the modification of yttrium oxide. 16 First, sintering temperature and speed affected the growth of crystal structure, material density, and pore shrinkage. 10 17 18 Increasing the firing temperature of monolithic zirconia resulted in enhanced translucency. 1 A firing temperature over 1,600°C was not recommended due to the reduction of flexural strength. 16 Extending the sintering time of YTZP significantly improves optical properties via the enlargement of grain and finally triggers the shift from the tetragonal to the monoclinic phase. 10 Rapid cooling of YTZP produced the larger grain size, as well as *t* - to *m* - phase transformation leading to the higher translucency while lowering flexural strength. 17 18 Second, the reduction and rearrangement of alumina at grain boundaries showed not only higher translucency but also higher strengths. 16 Third, the cubic structures were gained by increasing the yttrium oxide concentration (approximately 5 mol%) to become 5 mol% yttria-stabilized tetragonal polycrystalline (5YTZP), which contained approximately 50% cubic phase. 19 The cubic structures have a higher volume and are more isotropic so that the light scattering is less at the boundaries of grain, and the incident light is radiated more evenly in all directions, leading to better translucency of the material. 16 This also gave rise to aging-resistant YTZP ceramics 16 but sacrificed the flexural strength and fracture toughness, 19 since zirconia with a higher yttria concentration has a lower level of transformation toughening. 20

Recently, multilayered monolithic zirconia (Mu) has been manufactured by pressing various pigment-doped layers to mimic the gradual change of color from the cervical to the incisal of a human tooth. 21 A study reported that the color difference between layers was from pigment composition. 21 Moreover, some studies showed that adding coloring compounds can act as contaminants that impact the microstructure, translucency, 22 flexural strength, 19 and hardness. 23 For example, increasing ferric oxide (Fe _2_ O _3_ ) led to a material's enhanced hardness and higher CR, with a simultaneous loss of translucency. 24 Zirconia colored with erbium and neodymium ions resulted in a decrease in flexural strength and fracture toughness. 25 On the other hand, some reported that there was no significant difference in translucency 21 and flexural strength of different layers. 26 There are many reports on the adjustment of firing parameters such as heating speed, peak temperature, and sintered-holding time 10 16 ; however, there is a shortage of studies on the cooling speed. Moreover, monochrome (Mo) and multilayer (Mu) 5YTZP are currently a source of interest in modern dental practice, as they are being investigated in terms of aesthetic qualities when compared to other glass ceramics. The modification of sintering parameters, especially the cooling speed, was an essential solution for these materials, but there was not enough evidence to prove how cooling speed affects the optical properties of 5YTZP. This study determined the effect of varying sintering cooling rates on optical properties, including ∆ *E*_w,_ TP, CR, and OP of the monochrome and multilayer 5YTZP. The null hypotheses were no significant effect of varying sintered cooling rates, different type of materials, as well as their interactions on the optical properties of either Mo or Mu monolithic zirconias.

## Materials and Methods

The *in-vitro* investigation was performed with sample size estimation from Sailer et al 2007 27 using G*power 3.1 software (Heinrich-Heine-Universität, Düsseldorf, Germany) at the power of test = 0.9, and α error = 0.05 as in Equation (1). The number of sample sizes based on this calculation was 15 specimens per group used for this experiment.



Where: Z _α =_ standard normal deviation = 1.96 (α error = 0.05), Z _β =_ standard normal deviation = 1.28 (β error = 0.1), µ _1_ - µ _2_ = mean difference between groups = 0.8, and s = standard deviation (s _1_ =2.3, s _2_ = 1.5)

### Preparation Zirconia Specimens

Monochrome 5YTZP (Mo: Cercon xt, Dentsply Sirona, Charlotte, North Carolina, United States) and multilayer 5YTZP (Mu: Cercon xt ML, Dentsply Sirona, Charlotte, North Carolina, United States) were selected as presented in Table 1 . The material shade of A2 was chosen for all samples. The 5YTZP samples (Mo: monochrome, and Mu: multilayer) were cut ( *n* = 45/material, *N* = 90) using a wheel coated with diamond (Mecatome T180, PRESI France, Eybens, France). Silicon carbide abrasive paper from no. 320 up to no. 2000 was used to grind the bar specimens and then they were gently polished by 1 µm-fine diamond grain suspension using a polisher (Ecomet3, Beuhler, Illinois, United States) to obtain a specific size (width x length x thickness = 10 × 20 × 2 mm). The zirconia specimens were initially cut to an oversized bar (width x length x thickness = 12.5 × 25 × 2.5 mm) for shrinkage compensation after the firing procedure. After that, an ultrasonic cleaning device (Vitasonic II, Vita Zahnfabrik, Germany) was used to decontaminate the specimens in distilled water for 10 minutes, and then the materials were left to dry for an hour at room temperature. Monochrome and multilayer 5YTZP specimens were randomly allocated into different testing groups ( *n* = 15/group) according to three sintering modes, based on the range (minimum to maximum) of cooling rate alteration of the sintering furnace: slow cooling rate at 5°C/min (S), normal cooling rate at 35°C/min (N), and fast cooling rate at 70°C/min (F). The sintering program of both types of materials involved heating at 22 °C/min to 880 °C and then continuing to heat at 11 °C/min until reaching 1,500 °C, followed by cooling down to room temperature based on the aforementioned cooling parameters as categorized. A sintering mode was programmed on a ceramic furnace (inFire HTC, Dentsply Sirona, Charlotte, North Carolina, United States). The total sintering time for S-, N- and F- cooling rate protocol was 8 hours, 40 minutes; 4 hours, 30 minutes; and 4 hours, 10 minutes, respectively.

**Table 1:** Material, brand, material abbreviation (Abv.), manufacturers, batch number, and composition (wt%) of 5YTZP used in this study

Material	Brand	Abv.	Manufacturer	Batch no.	Composition (%Weight)
5YTZP (Monochrome)	Cercon xt	Mo	Cercon xt, Dentsply Sirona, Charlotte, North Carolina, United States	18040682	≥99% ZrO _2_ + HfO _2_ + Y _2_ O _3,_ 9% Y _2_ O _3,_ <3% HfO _2_ <1% Al _2_ O _3,_ + SiO _2_
5YTZP (Multilayer)	Cercon xt ML	Mu	Cercon xt, Dentsply Sirona, Charlotte, North Carolina, United States	18041981, 18042302	≥99% ZrO _2_ + HfO _2_ + Y _2_ O _3,_ 9% Y _2_ O _3,_ <3% HfO _2_ <1% Al _2_ O _3,_ + SiO _2_

Abbreviation: 5YTZP, 5mol% yttria-stabilized tetragonal zirconia polycrystalline.

### Determination Optical Parameters

A laboratory spectrophotometer (ColorQuest XE, Hunter Associates Laboratory, Reston, Virginia, United States) was used to measure the optical properties of monochrome 5YTZP and cervical and incisal halves of multilayer 5YTZP in different cooling protocols. The instrument settings were controlled at illuminant D65, 10% observer angle, 100% ultraviolet, a standard wavelength of 380 to 780 nm, and a 4 mm diameter of the aperture. The CIEL*a*b* (Commission International de I'Eclairage) was determined. To calibrate the machine before testing, a standard white tile (L* = 96.7, a* = 0.1, b* = 0.2) was used. Moreover, to maintain the specimen position and measuring points on the cervical and incisal layers, a clear jig was used. The L*, a*, and b* values were then calculated for color appearance (∆ *E*_w_ ), color appearance difference (∆ *E*_diff_ ), TP, CR, and OP. The ∆ *E*_w_ and ∆ *E*_diff_ were achieved from lightness (  ), the green-red coordinate (  ), and the blue-yellow (  ) coordinate of specimens on a white background, and the coordinate of VITA classic shade A2 (  ) 28 according to Equation (2), and Equation (3). 10 13 17





The color differences between color determinants on black (L* = 10.4, a* = 0.4, b* = 0.6) and white (L* = 96.7, a* = 0.1, b* = 0.2) backgrounds were used to calculate the TP values, according to Equation (4). 13



The CR values were calculated using Equations (5) and (6). 13 The CR values are between 0 (transparent) and 1 (completely opaque); Y is the luminance according to Tristimulus Color Space/XYZ; *Y*_B_ is the value of a specimen recorded on a black background; *Y*_w_ is the value of a specimen recorded on a white background; *Y*_n_ is equal to 100.





The OP values were determined using Equation (7) below. 13



### Determining the Microstructure and Chemical Composition

At a vacuum of 130 m torr and a current of 10 mA, gold was applied to the specimens for 3 minutes, and then a desiccator cabinet was used for drying the specimens. The grain and microstructure were assessed using a scanning electron microscope (Hitachi S-300N, Osaka, Japan). Additionally, the chemical composition of each material was analyzed by energy dispersive spectroscopy (EDS).

### Determination of the Phase Percentage

An X-ray diffractometer (PANalytical, Empyrean, Almelo, the Netherlands) was used to determine the amount of crystal structure of zirconia by their relative proportions. Utilizing copper k-alpha (Cu Kα) radiation, the scanning of specimens was performed at diffraction angles (2θ) of 20 to 80 degrees with 0.02 degrees step size at intervals of two seconds. By cross-referencing with the database of the Joint Committee of Powder Diffraction Standards, the zirconia phase was examined. A software package (X'Pert Plus, Philips, Almelo, the Netherlands) was used to analyze the relative proportions of phases by peak intensity. The monoclinic, tetragonal, and cubic phases' peaks were identified using PDF files Nos. 37-1484, 49-1642, and 42-1164, respectively. The proportion of monoclinic ( *X*_m_ ) was estimated using the Garvie-Nicholson formula, as shown in Equation 8. 29 As the tetragonal and cubic (111) peaks cannot be distinguished, the sum of (004)t and (220)t tetragonal peaks and (400)c cubic peak were used to calculate the variations in the proportions of tetragonal ( *X*_t_ ) and cubic ( *X*_c_ ) phases, as shown in Equations (9) and (10). 30







The integrated intensities for cubic, tetragonal, and monoclinic are shown in *I*_c_ , *I*_t_ , and *I*_m_ , respectively. These were calculated by matching the complementary peaks with a pseudo-Voigt distribution and investigating the area under the curves. The nonlinear calibration curve of integrated intensity ratios versus volume fraction was used to estimate a correction factor of 1.311 in Equation 8, to account for the effect of yttria doping on the lattice parameters.

### Statistical Analysis

The significant differences in optical parameters of monochrome and multilayer 5YTZP that were subjected to various cooling protocols were determined using two-way analysis of variance (ANOVA) and post hoc Bonferroni multiple comparison techniques within a statistical software package (IBM SPSS Statistics 20, SPSS Inc., Chicago, Illinois, United States). At *p* < 0.05, a result was deemed statistically significant. Descriptive analysis was also performed to determine the optical parameters, grain size, chemical composition, and phase percentage of zirconia.

## Results

**Fig. 1:**
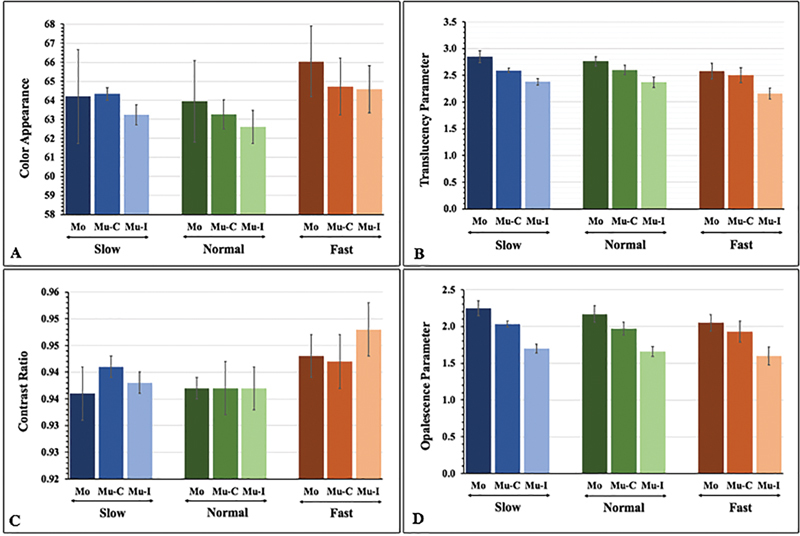
Color appearance ( **A** ), translucency parameter ( **B** ), contrast ratio ( **C** ), and opalescent parameter ( **D** ) of monochrome (M), multilayer-cervical (Mu-C), and multilayer-incisal (Mu-I) 5 mol% yttria-stabilized tetragonal polycrystalline upon slow- (S), normal- (N), and fast- (F) cooling protocols.

The optical parameters (color appearance, TP, CR, and OP) for each testing group, along with their mean, standard deviation, and 95% confidence interval (95% CI) are presented in Table 2 and Fig. 1 . The Δ *E*_W_ was the highest in MoF (66.04 ± 1.86), followed by MuF-C (64.72 ± 1.49), and MuF-I (64.59 ± 1.24), while the lowest Δ *E*_W_ was in MuN-I (62.60 ± 0.86). The TP was the highest in MoS (2.85 ± 0.11), followed by MoN (2.76 ± 0.09), and MuS-C (2.59 ± 0.04), whereas the lowest TP was in MuF-I (2.16 ± 0.10). The CR was the highest in MuF-I (0.948 ± 0.005), followed by MoF (0.943 ± 0.004), and MuF-C (0.942 ± 0.005), whereas the lowest CR was in MoS (0.936 ± 0.005). The OP was the highest in MoS (2.25 ± 0.10), followed by MoN (2.17 ± 0.11), and MoF (2.05 ± 0.11), while the lowest OP was in MuF-I (1.60 ± 0.12). The ∆ *E*_diff_ was the highest in MoF (3.83) followed by MuF-C (2.51), and MuF-I (2.38), whereas the lowest ∆ *E*_diff_ was in MuN-I (0.39). Statistically significant differences in Δ *E*_W_ , TP, CR, and OP caused by a differing type of zirconia, cooling rates, and their interactions (p < 0.05) were revealed by 2-way ANOVA. Nevertheless, there was no significant difference in the interactions between Δ *E*_W_ and OP, as presented in Table 3 .

**Table 2:** Mean, standard deviation (SD), 95% confidential interval (CI), color appearance (ΔΕ), translucency parameter (TP), contrast ratio (CR), and opalescent parameter (OP), color difference (Δ *Ε*_diff_ ), relative phase content (wt.%) and average grain size (nm), of monochrome (M), multilayer-cervical (Mu-C), and multilayer-incisal (Mu-I) 5YTZP upon slow- (S), normal- (N), and fast- (F) cooling protocols

Group	∆ *E*_W_ Mean ± SD (95%CI)	TP Mean ± SD (95%CI)	CR Mean ± SD (95%CI)	OP Mean ± SD (95%CI)	∆ *E*_diff_	Relative phase (wt%)			Grain size (Mean ± SD)
						*m* -	*t* -	*c* -	
MoS	64.20 ± 2.46 (62.83–65.56)	2.85 ± 0.11 (2.79–2.91)	0.936 ± 0.005 (0.933–0.938)	2.25 ± 0.10 (2.20–2.31)	1.99	12.96	55.94	31.10	1046.16 ± 383.48
MuS-C	64.33 ± 0.34 (64.15–64.52)	2.59 ± 0.04 (2.56–2.61)	0.941 ± 0.002 (0.940–0.942)	2.03 ± 0.04 (2.01–2.06)	2.12	13.56	55.64	30.80	1028.23 ± 325.48
MuS-I	63.23 ± 0.52 (62.94–63.52)	2.38 ± 0.06 (2.34–2.41)	0.938 ± 0.002 (0.937–0.940)	1.70 ± 0.06 (1.66–1.73)	1.02	13.09	57.68	29.23	1018.30 ± 419.32
MoN	63.94 ± 2.14 (62.76–65.12)	2.76 ± 0.09 (2.71–2.81)	0.937 ± 0.002 (0.936–0.939)	2.17 ± 0.11 (2.11–2.23)	1.73	12.82	55.64	31.54	1035.94 ± 410.39
MuN-C	63.26 ± 0.76 (62.84–63.68)	2.60 ± 0.09 (2.55–2.65)	0.937 ± 0.005 (0.935–0.940)	1.97 ± 0.09 (1.92–2.02)	1.05	13.62	55.50	30.88	1035.94 ± 368.17
MuN-I	62.60 ± 0.86 (62.13–63.08)	2.37 ± 0.10 (2.31–2.42)	0.937 ± 0.004 (0.935–0.939)	1.66 ± 0.07 (1.62–1.70)	0.39	13.06	57.96	28.98	1017.71 ± 454.20
MoF	66.04 ± 1.86 (65.01–67.07)	2.58 ± 0.15 (2.50–2.66)	0.943 ± 0.004 (0.941–0.946)	2.05 ± 0.11 (1.98–2.11)	3.83	13.30	55.93	30.77	994.15 ± 474.86
MuF-C	64.72 ± 1.49 (63.89–65.54)	2.50 ± 0.14 (2.42–2.58)	0.942 ± 0.005 (0.939–0.945)	1.93 ± 0.14 (1.86–2.01)	2.51	14.06	56.17	29.77	982.52 ± 407.08
MuF-I	64.59 ± 1.24 (63.90–65.28)	2.16 ± 0.10 (2.10–2.21)	0.948 ± 0.005 (0.945–0.950)	1.60 ± 0.12 (1.54–1.67)	2.38	13.25	57.91	28.84	990.01 ± 348.00

Abbreviation: 5YTZP, 5mol% yttria-stabilized tetragonal zirconia polycrystalline.

**Table 3:** Two-way ANOVA of color appearance (A), translucency parameter (B), contrast ratio (C), and opalescent parameter (D) of monochrome and multilayer 5YTZP upon different cooling protocols

(A) ANOVA of color appearance of 5YTZP upon different cooling protocols					
Source	SS	df	MS	F	* p* -Value
Corrected model	122.274	8	15.284	7.039	< 0.001
Intercept	554704.971	1	554704.971	255472.247	< 0.001
layerMat	35.096	2	17.548	8.082	< 0.001
Protocol	78.815	2	39.408	18.149	< 0.001
layerMat * Protocol	8.363	4	2.091	0.963	0.430
Error	273.583	126	2.171		
Total	555100.828	135			
**(B) ANOVA of translucency parameter of 5YTZP upon different cooling protocols**					
** Source**	** SS**	** df**	** MS**	** F**	*** p*** **-Value**
Corrected model	5.373	8	0.672	62.165	< 0.001
Intercept	865.439	1	865.439	80102.277	< 0.001
layerMat	4.280	2	2.140	198.055	< 0.001
Protocol	0.938	2	0.469	43.417	< 0.001
layerMat * Protocol	0.155	4	0.039	3.593	0.008
Error	1.361	126	0.011		
Total	872.174	135			
**(C) ANOVA of contrast ratio of 5YTZP upon different cooling protocols**					
** Source**	** SS**	** df**	** MS**	**F**	***p*** **-Value**
Corrected model	0.002	8	0.000	13.832	<0.001
Intercept	119.288	1	119.288	7541099.597	< 0.001
layerMat	0.000	2	5.794E-5	3.663	0.028
Protocol	0.001	2	0.001	40.241	< 0.001
layerMat * Protocol	0.000	4	9.037E-5	5.713	< 0.001
Error	0.002	126	1.582E-5		
Total	119.292	135			
**(D) ANOVA of opalescence parameter of 5YTZP upon different cooling protocols**					
** Source**	** SS**	** df**	** MS**	**F**	***p*** **-Value**
Corrected model	6.272	8	0.784	81.501	<0.001
Intercept	502.953	1	502.953	52280.451	< 0.001
layerMat	5.807	2	2.904	301.830	< 0.001
Protocol	0.398	2	0.199	20.697	< 0.001
layerMat * Protocol	0.067	4	0.017	1.738	0.146
Error	1.212	126	0.010		
Total	510.438	135			

Abbreviations: 5YTZP, 5mol% yttria-stabilized tetragonal zirconia polycrystalline; ANOVA, analysis of variance; df, degree of freedom; F, F-ratio; MS, mean square; SS, sum of squares.

**Fig. 2:**
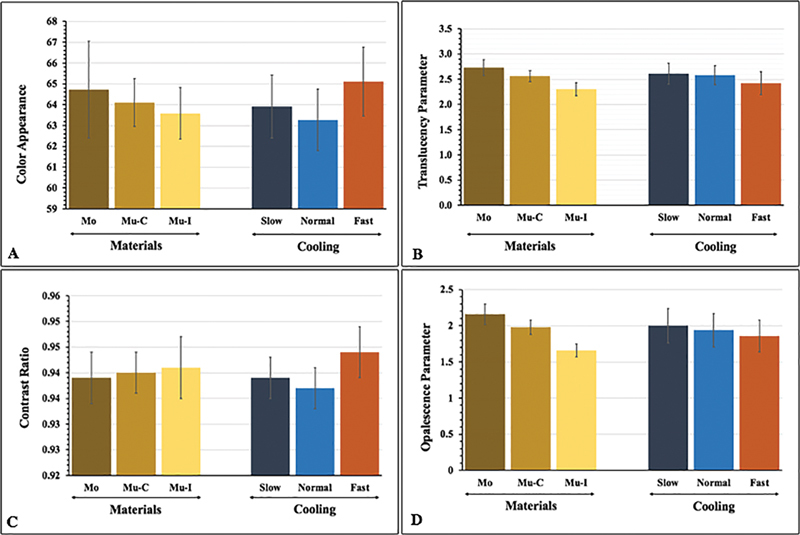
Color appearance ( **A** ), translucency parameter ( **B** ), contrast ratio ( **C** ), and opalescent parameter ( **D** ) of monochrome (M), multilayer-cervical (Mu-C), and multilayer-incisal (Mu-I) 5 mol% yttria-stabilized tetragonal polycrystalline and slow- (S), normal- (N), and fast- (F) cooling protocols.

Bonferroni post hoc comparisons showed that different types of zirconia reveal significant differences ( *p* < 0.05) in Δ *E*_W_ , TP, CR, and OP, except between group Mo/Mu-C in CR, as shown in Table 4 and Fig. 2 . Moreover, the statistical analysis depicted that different cooling rates had significant differences ( *p* < 0.05) in Δ *E*_W_ , TP, CR, and OP, except between group S/N in TP and CR, as presented in Table 4 and Fig. 2 . Additionally, they showed an interaction of different types of materials, and varied cooling protocols showed significant differences in the TP ( *p* < 0.05), except for MoS/MoN, MuS-C/MuN-C, MuS-C/MoF, MuS-C/MuF-C, MuS-I/MuN-I, MuS-I/MuF-C, MuN-C/MoF, MuN-C/MuF-C, MuN-I/MuF-C, and MoF/MuF-C group, and significant difference in CR (p < 0.05) except for the MoS/MuS-I, MoS/MoN, MoS/MuN-C, MoS/MuN-I, MuS-C/MuN-C, MuS-C/MuN-I, MuS-C/MoF, MuS-C/MuF-C, MuS-I/MoN, MuS-I/MuN-C, MuS-I/MuN-I, MuS-I/MuF-C, MoN/MuN-C, MoN/MuN-I, MoN/MuF-C, MuN-C/MuN-I, MuN-C/MuF-C, MuN-I/MuF-C, MoF/MuF-C, MoF/MuF-I, and MuF-C/MuF-I groups as presented in Table 4 and Fig. 1 .

**Table 4:** Post hoc Bonferroni multiple comparisons of color appearance (A), translucency parameter (B), contrast ratio (C), and opalescent parameter (D) of monochrome (Mo), multilayer-cervical (Mu-C), and multilayer-incisal (Mu-I) 5YTZP upon slow- (S), normal- (N), and fast- (F) cooling protocols

(A) Post hoc of color appearance as a function of material types and cooling protocols									
Material		Mo	Mu-C	Mu-I	Cooling		S	N	F
**Mo**		1	0.048	<0.001	**S**		1	0.038	<0.001
**Mu-C**			1	0.045	**N**			1	<0.001
**Mu-I**				1	**F**				1
**(B) Post hoc of translucency parameter as a function of material types and cooling protocols**									
**Material**		**Mo**	**Mu-C**	**Mu-I**	**Cooling**		**S**	**N**	**F**
**Mo**		1	<0.001	<0.001	**S**		1	0.187	<0.001
**Mu-C**			1	<0.001	**N**			1	<0.001
**Mu-I**				1	**F**				1
**(C) Post hoc of contrast ratio as a function of material types and cooling protocols**									
**Material**		**Mo**	**Mu-C**	**Mu-I**	**Cooling**		**S**	**N**	**F**
**Mo**		1	0.101	0.008	**S**		1	0.128	<0.001
**Mu-C**			1	0.305	**N**			1	<0.001
**Mu-I**				1	**F**				1
**(D) Post hoc of opalescence parameter as a function of material types and cooling protocols**									
**Material**		**Mo**	**Mu-C**	**Mu-I**	**Cooling**		**S**	**N**	**F**
**Mo**		1	<0.001	<0.001	**S**		1	0.005	<0.001
**Mu-C**			1	<0.001	**N**			1	<0.001
**Mu-I**				1	**F**				1
**(E) Post hoc of translucency parameter among groups of different materials with differed cooling protocol**									
**Groups**	**MoS**	**MuS-C**	**MuS-I**	**MoN**	**MuN-C**	**MuN-I**	**MoF**	**MuF-C**	**MuF-I**
**MoS**	1	<0.001	<0.001	0.576	<0.001	<0.001	<0.001	<0.001	0.000
**MuS-C**		1	<0.001	<0.001	1	<0.001	1	0.831	<0.001
**MuS-I**			1	<0.001	<0.001	1	0.002	0.160	<0.001
**MoN**				1	0.002	<0.001	0.021	<0.001	<0.001
**MuN-C**					1	<0.001	1	0.769	<0.001
**MuN-I**						1	0.002	0.181	<0.001
**MoF**							1	0.996	<0.001
**MuF-C**								1	<0.001
**MuF-I**									1
**(F) Post hoc of contrast ratio among groups of different materials with differed cooling protocol**									
**Groups**	**MoS**	**MuS-C**	**MuS-I**	**MoN**	**MuN-C**	**MuN-I**	**MoF**	**MuF-C**	**MuF-I**
**MoS**	1	0.021	0.888	1	1	1	0.003	0.043	<0.001
**MuS-C**		1	0.014	0.001	0.232	0.064	0.983	1	0.013
**MuS-I**			1	0.998	1	1	0.025	0.409	<0.001
**MoN**				1	1	1	0.003	0.085	<0.001
**MuN-C**					1	1	0.038	0.318	<0.001
**MuN-I**						1	0.012	0.165	<0.001
**MoF**							1	1	0.524
**MuF-C**								1	0.160
**MuF-I**									1

**Fig. 3:**
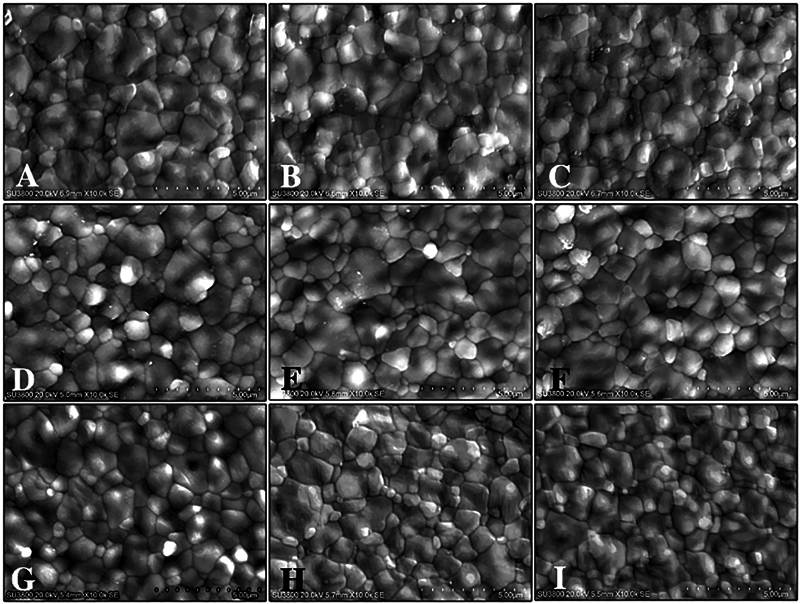
Scanning electron microscope image indicated grain size and grain distribution of monochrome ( **A** , **D** , **G** ) multilayer cervical ( **B** , **E** , **H** ) and multilayer incisal ( **C** , **F** , **I** ) zirconia upon slow- ( **A** , **B** , **C** ), normal- ( **D** , **E** , **F** ), and fast- ( **G** , **H** , **I** ) cooling protocols, at x10K magnification

**Fig. 4:**
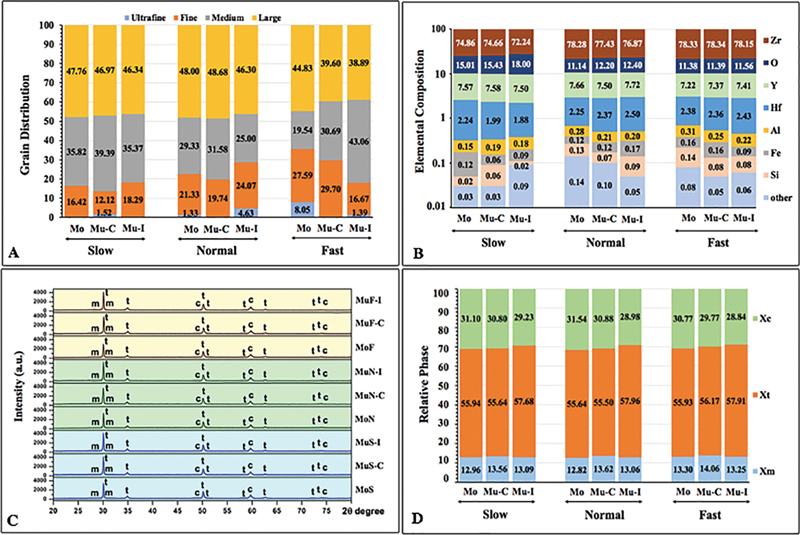
Grain distribution ( **A** ), elemental composition ( **B** ), x-ray diffraction ( **C** ), and relative phase ( **D** ) of monochrome ( **M** ), multilayer-cervical (Mu-C), and multilayer-incisal (Mu-I) 5 mol% yttria-stabilized tetragonal polycrystalline upon slow- (S), normal- (N), and fast- (F) cooling protocols.

The micrograph of monochrome and multilayer 5YTZP are shown in Fig. 3 . Four-grain sizes were used to define the zirconia crystal structures: ultrafine (≤400 nm), fine (400 < x≤700 nm), medium (700 < x≤1000 nm), and large (>1000 nm). The distribution (%) of four-grain size was presented in Fig. 4 . The amount of large grain tended to decrease with the increase in cooling rate. The average grain size (nm) of MoS was the largest (1046.16 ± 383.48), followed by MoN (1035.94 ± 410.39) and MuN-C (1035.94 ± 368.17), whereas the MuF-C was the smallest (982.52 ± 407.08), as shown in Fig. 4 and Table 2 . All zirconia groups indicated crystal structures mostly of large grains, except for MuF-I, which showed a majority of medium to large size. A fast-cooling rate resulted in limited grain growth and displayed smaller grain compared to slow and normal cooling rates. The composition analysis of all groups revealed the major elements of zirconia, oxygen, and yttrium, as presented in Fig. 4 .

X-ray diffraction analysis of the specimens' microstructure is shown in Fig. 4 . The x-ray diffraction patterns illustrated a majority of the tetragonal phase, followed by the cubic and monoclinic phases. The tetragonal phase was revealed at a diffraction angle of 30, 34.86, 73.2, and 74.2 degrees. The cubic phase was observed at 74.7 degrees, and the monoclinic phases were observed at 28 and 31.2 degrees. Concentrations with weight percentage (wt%) of monoclinic ( *X*_m_ ): tetragonal ( *X*_t_ ): cubic ( *X*_c_ ) phases were relatively demonstrated as shown in Fig. 4 . The cooling speed was related to the proportion of the zirconia phase. In a group with a faster cooling protocol, the proportion of the monoclinic phase increased.

## Discussion

To achieve restorations with a natural character, better translucency, as well as enhanced opalescence-of Mo and Mu 5YTZP, modifying the cooling speed of the sintering process was investigated in this study. The statistically significant differences in all optical parameters of different types of zirconia with varied cooling rates, as well as their interactions, were found, except for the interaction in ∆ *E*_w_ , and OP. Hence, all null hypotheses were partially rejected. In this study, the color appearance of the A2 color of the VITA Classical shade was 62.21, which was used as a reference. A CIE-based color perception system classified the color appearance difference ∆ *E*_diff_ as follows: the ∆ *E*_diff_ indicated “clinically indifferent” as ∆ *E*_diff_ < 3, “clinically acceptable” as ∆E = 3–5, and “clinically unacceptable” as ∆ *E*_diff_ > 5. 11 All Cercon xt and Cercon xt ML were considered “clinically indifferent” except the Cercon xt with a fast protocol (MoF), which was interpreted as “clinically acceptable,” as ∆ *E*_diff_ = 3.83. Regarding the color selection by VITA shade, the normal cooling protocol illustrated the lowest ∆ *E*_diff_ value, while the fast protocol of all materials presented the highest ∆ *E*_diff_ value. This implied that both monochrome and multilayer 5YTZP were suggested for the normal cooling protocol to optimally match with the VITA shade tab. Moreover, the incisal layer presented a lesser ∆ *E*_diff_ value than the cervical layer and monochrome material. This suggested that the incisal layer of multilayer 5YTZP could be the representative area of color matching to the VITA shade.

Concerning translucency, a crucial property to simulate the natural character and appearance of tooth structure, particularly in the aesthetic region. 31 This could be described by TP and CR values. This study showed significant differences in TP among each pair of material groups. The TP value of Mo was the highest, followed by Mu-C, and Mu-I. On the other hand, the CR value of Mu-I was the highest, followed by Mu-C and Mo. This implied that the monochrome 5YTZP had better translucency than the multilayer 5YTZP. Although the manufacturer's data sheet showed no difference in the major components of both materials, some found that the only difference between material layers was the colorant additives, which might lead to a difference in translucency. 21 Moreover, the EDS analysis showed that the amount of elemental composition was in the same range among monochrome and multilayer 5YTZP. Previous studies mentioned that the firing parameter alteration affected the growth of crystal structure, material density, and pore shrinkage. 10 17 18 There was no recent evidence proving that there was an elemental transition due to the cooling parameter alteration. The minor difference in the chemical composition of 5YTZP upon various cooling speeds might be due to the dispersion of elements on a different field of view investigated by EDS analysis. Further study on this investigation should be accomplished.

As evidenced by a lower TP value, increasing the cooling rate significantly diminished the translucency of monochrome and multilayer 5YTZP. Insufficient grain growth of crystal contents probably played a role, as observed in limited grain enlargement in the fast-cooling group. The fine grains resulted from the limited grain enlargement, presented the numerous grain boundaries that induced the scattering of light on it, meaning that the remaining light passing through the material decreased, and consequently led to the reduction of translucency. This finding was consistent with previous studies showing that smaller grains and limited grain growth led to a drop in the translucency of YTZP. 10 17 18 This could be evidenced that, to enhance the translucency of stabilized zirconia, the enlargement of grain was required. Moreover, this rapid temperature change triggers stress within the zirconia structure and consequently leads to a t-m phase transformation 10 as supported by an increase in the monoclinic phase. The increase in monoclinic phase in the fast-cooling protocol produced more significant light scattering, due to the different refractive indices of the crystal structures, compared to zirconia with a majority of cubic and tetragonal phases and minute monoclinic content. Moreover, the sintering process is the phenomenon of a growing grain of zirconia, increasing the density, as well as reducing the porosity. Extending sintering time by reducing the cooling rate may lead to more homogeneity of the crystal structure, which promotes better light transmission. As a result, this study provides evidence that decreasing the cooling speed may achieve increased translucency. However, the strength of the material should be concerned for clinical application according to the cooling effect. There was a study showing that an increase in grain sizes and t- to m- phase transformation negatively resulted in the flexural strength of 3YTZP. 18 Since the published study regarding cooling speed alteration was a shortage, further investigation on the mechanical properties of 5YTZP should be conducted.

The OP was also investigated. The materials with an OP value closer to the tooth enamel were a goal for tooth appearance simulation. The OP was significantly different among the layers of the material. The mean OP of the testing groups was 1.60 to 2.25, which was less than that of human enamel (19.8–27.6) 15 The higher the speed of the sintering process, the lower the OP value. Additionally, the mean OP of the incisal layer was less than that of the cervical layer and monochrome material. This might be due to the pigment additives, which affect the light trajectory, and opalescence phenomenon.

To summarize, both Cercon xt and Cercon xt ML were suggested for slow (5°C/min) and normal cooling protocols (35°C/min) to achieve the most favorable translucency and opalescence, which was slower than the manufacturer recommendation protocol at 99°C/min. Increasing cooling speed resulted in smaller grain size, t-m transformation, and finally lower translucency and opalescence. Moreover, the incisal layer of multilayer 5YTZP was perfectly matched with VITA shade. Apart from the translucency, the strength of each material will be further studied to prove which protocol is suitable in optimizing both optical and mechanical properties. These results provided data on the effect of modifying sintering parameters to optimize the optical properties of monochrome and multilayer zirconia. This study supports the proposal that to obtain better translucency and opalescence of 5YTZP, the sintering cooling rate should be reduced.

## Conclusion

The study herein revealed that the ∆ *E*_W_ , TP, CR, and OP of monochrome and multilayer 5YTZP were affected by the type of zirconia, cooling rate of the sintering process, and their interaction, except the interaction for ∆ *E*_W_ and OP. The translucency of monochrome 5YTZP, cervical, and incisal layers of multilayer 5YTZP was different, possibly due to the colorant additives. The incisal layer of multilayer 5YTZP was perfectly matched with the VITA shade tab, having the least color appearance difference value. Increasing the cooling speed of 5YTZP resulted in smaller grain size as well as t-m transformation, and finally led to lower translucency and opalescence and increased opacity. Therefore, to achieve the most favorable optical properties, a slow cooling rate was recommended.
